# The influence of social relationship on food tolerance in wolves and dogs

**DOI:** 10.1007/s00265-017-2339-8

**Published:** 2017-06-30

**Authors:** Rachel Dale, Friederike Range, Laura Stott, Kurt Kotrschal, Sarah Marshall-Pescini

**Affiliations:** 1 Comparative Cognition, Messerli Research Institute, University of Veterinary Medicine, Medical University of Vienna, University of Vienna, 1 Veterinaerplatz, 1210 Vienna, Austria; 2 Wolf Science Center, Messerli Research Institute, University of Veterinary Medicine, Vienna, Austria; 3 Department of Behavioural Biology, University of Vienna, Vienna, Austria

**Keywords:** Canid, Food tolerance, Social relationship, Feeding context

## Abstract

**Abstract:**

Food sharing is relatively widespread across the animal kingdom, but research into the socio-ecological factors affecting this activity has predominantly focused on primates. These studies do suggest though that food tolerance is linked to the social relationship with potential partners. Therefore, the current study aimed to assess the social factors which influence food tolerance in two canids: wolves and dogs. We presented wolves and dogs with two paradigms: dyadic tolerance tests and group carcass feedings. In the dyadic setting, the affiliative relationship with a partner was the most important factor, with a strong bond promoting more sharing in both species. In the group setting, however, rank was the primary factor determining feeding behavior. Although the dominant individuals of both species defended the carcass more than subordinates, in the dogs, the subordinates mostly stayed away from the resource and the most dominant individual monopolized the food. In the wolves, the subordinates spent as much time as dominant individuals in proximity to, and feeding from, the carcass. Furthermore, subordinate wolves were more able to use persistence strategies than the dogs were. Feeding interactions in the wolves, but not dogs, were also modulated by whether the carcass was on the ground or hanging from a tree. Overall, the social relationship with a partner is important in food distribution in wolves and dogs, but the precise effects are dependent on species and feeding context. We consider how the different socio-ecologies of the two species may be linked to these findings.

**Significance statement:**

Despite the fact that food sharing is relatively widespread in the animal kingdom, the specific factors underlying whether an animal will share with a specific individual are little understood. When it comes to decisions about food sharing in wolves and dogs, friendship is the deciding factor if it is just two of you, but in a bigger group rank position decides your access to the spoils. What is more, it seems that rank positioning is even more important in dogs than wolves as dominant dogs keep the food for themselves while each wolf pack member has a chance to eat. This is the first evidence that the importance of the social relationship in food sharing is dependent on the feeding context in canids.

**Electronic supplementary material:**

The online version of this article (doi:10.1007/s00265-017-2339-8) contains supplementary material, which is available to authorized users.

## Introduction

Food sharing is generally defined as the joint use of a monopolizable food resource by more than one individual (Stevens and Gilby 2004) or the transfer of a food item from one individual to another (Feistner and McGrew 1989). Food sharing incurs a cost to the food possessor and, as such, is the most commonly seen prosocial behavior in non-human animals, with evidence of its occurrence in a wide variety of species (e.g., Clutton-brock 1991; Vahed 1998; Carter and Wilkinson 2015). However, although food sharing has been observed in many species, the mechanisms and principles regulating an individual’s choice to share food with another are still under debate. The primary hypotheses proposed thus far are as follows: (1) kin selection: whereby animals provision offspring or those closely related (Feistner and McGrew 1989); (2) reciprocity: where an individual relinquishes food to another in exchange for either past or future benefits from the recipient such as receiving food, grooming, sex, or support in conflict (Brosnan and de Waal 2002); and (3) harassment avoidance: when a possessor gives up a resource because this holds a lower cost than defending it or refusing such solicitation would incur (Gilby 2006).

In addition to these hypotheses, it has recently been suggested that the quality or type of the relationship shared with another individual may also be an important factor in tolerance around food sources. Jaeggi and van Schaik (2011) suggested that food sharing when under pressure (i.e., when being solicited to do so by the potential receiver) is inextricably linked to the social relationship between the individuals. Rejecting this harassment may have more social costs at certain times or with certain individuals than others; for example, rejecting the harassment of friends or potential mates may lead to loss of future reciprocal benefits. Therefore, they argue that harassment avoidance can explain the general occurrence of food sharing, but additional explanations are often required to account for the specific possessor-recipient combinations seen to engage in this activity.

Following this, some authors have proposed that food sharing may include a “social assessment” aspect. Specifically, hypotheses have been proposed to suggest that beggars are often not primarily interested in food but rather use the response of the possessor to ascertain information about their personality (the “information gathering” hypothesis; van Noordwijk and van Schaik 2009) or about the status of their relationship with them (the “assessing-relationships” hypothesis; Goldstone et al. 2016). Related to this, some have suggested that food sharing can in fact be used not only to assess, but also to establish or reinforce social bonds (von Bayern et al. 2007; Wittig et al. 2014; Yamamoto 2015). Yamamoto (2015) observed that the reciprocity and harassment avoidance hypotheses mainly stem from meat sharing in chimpanzees, a highly sought after commodity in a competitive species. Bonobos on the other hand share abundant fruits that individuals are able to gain individually, suggesting that reasons other than nutritional gain may be at play (see also Slocombe and Newton-Fisher 2005).

Although it is still a limited field of research, a growing body of empirical evidence over the last few years does support this more social view of food sharing, at least in primates. A strong affiliative bond has been shown to be an important factor in food tolerance both in wild and captive populations. For instance, co-feeding patterns significantly correlated with grooming relationships, but not genetic relatedness, in Chacma baboons (King et al. 2011). Although they could not rule out an impact of kinship entirely due to the small number of highly related adult dyads, the results do indicate an importance of a strong social bond in co-feeding tolerance. In a captive setting, where more factors can be controlled, including food distribution and knowledge of genetic and social relationships, chimpanzees that were highly affiliated with a food possessor were more likely to receive food than those that typically avoided the possessor in a group of female chimpanzees (Eppley et al. 2013). Although females shared a higher proportion of food with some kin than non-kin members, they only did so when they also shared a close affiliative bond with the related individual. These results were mediated by perseverance, with closely bonded partners showing more “begging” behaviors than partners with less affiliative bonds, suggesting that in female chimpanzees, persistently requesting food is only possible if you have a good relationship with the possessor.

There is some evidence suggesting that the story is not as simple as animals providing more food to “friends,” as in some studies this effect is additionally mediated by rank. For example, although grooming predicted food sharing in the study by King et al. (2011), dominant baboons were central to the feeding network, meaning they frequently shared food patches with multiple group members, thus giving these individuals more opportunity to demonstrate sharing with particular individuals. Similarly, using a long-term data set, O’Malley et al. (2016) recently found that high-ranking wild female chimpanzees spent more time eating meat than low-ranking females. In chimpanzees, meat is acquired by the males and shared with other group members, suggesting that males share more meat with high-ranking females. No such rank difference in female feeding was found in the consumption of insects, which can be acquired individually. In addition, although wild Japanese macaques spent more time co-feeding with females which groomed them the most, this relationship became even stronger when the analysis was restricted to grooming directed from low to high ranking animals (Ventura et al. 2006). This suggests that the low-ranked individuals may use grooming to “buy” the food tolerance of dominant animals.

Despite the fact that food sharing occurs in a wide variety of species, it is clear that the investigations into the factors affecting food sharing have predominantly focused on primates. Although some studies have mentioned the potential influence of hierarchy on feeding behavior of non-primates (Hirsch 2007; black bears: Rogers 1987; cats: Bonanni et al. 2007; red deer: Appleby 1980; pigs: Held et al. 2010; rooks: Scheid et al. 2008; hyenas: Smith et al. 2007), comprehensive assessments of how social relationships influence feeding in other species remain lacking. Therefore, the current study aimed to assess the conditions which promote, or inhibit, food sharing in a non-primate taxon, i.e., canids. Canids are particularly interesting because many species, particularly those reliant on cooperative hunting, share food on a much more regular basis than primates (Moehlman 1989); yet, almost nothing is known about how they negotiate the distribution of food among group members.

Specifically, gray wolves (*Canis lupus*) and domestic dogs (*Canis lupus familiaris*) were our model species since they diverged relatively recently; yet, their social organization and feeding ecologies are considerably different. Domestic dogs originally derive from ancestral gray wolves (Lindblad-toh et al. 2005; Frantz et al. 2016), and both species are highly social, forming stable social bonds with group members over multiple years (Harrington and Mech 1982; Bonanni et al. 2010). Wolves typically live in monogamous family units consisting of a breeding pair and the offspring from previous years (Mech and Boitani 2003). They rely on cooperative hunting and breeding (Mech and Boitani 2003; MacNulty et al. 2012), and as such, food sharing is an essential part of the survival of pack members, via distribution of large prey brought down together and regurgitation of food by adults for puppies (Mech et al. 1999). In comparison, free-ranging dogs (whose movements, activities, and reproduction are not constrained by humans; Cafazzo et al. 2014) live in promiscuous packs, and although capable of hunting (Manor and Saltz 2004; Silva-Rodríguez and Sieving 2012), they seem to rely more on individual scavenging, predominantly on human refuse, as their primary foraging technique (Butler et al. 2004; Vanak and Gompper 2009a).

These differences in foraging techniques may be responsible for recently observed differences in food tolerance between the two species (Marshall-Pescini et al. 2017). In our similarly raised and kept sample of captive wolves and dogs, where direct comparisons between wolves and dogs are possible, it has been shown that dogs exhibit more despotic tendencies around food, whereby the dominant individual monopolizes the resource, whereas subordinate wolves were able to challenge their partners for access to the food (Range et al. 2015). These findings are in line with long-term wild wolf research by Mech (1999), who found that regardless of rank, each wolf is able to defend their food source. Furthermore, in a free-ranging dog population, Cafazzo et al. (2010) found that around food, adult high-ranking dogs showed most aggression to middle-ranking dogs and relatively little to low-ranking individuals. Overall, this suggests that rank is a particularly relevant factor in mediating food tolerance in dogs, but potentially less so in wolves. Furthermore, we have recently shown that commodity exchange is relevant in discriminate food sharing, with both species, but particularly dogs, showing a food-for-sex effect, adapting their behavior around food depending on female reproductive stage (Dale et al. 2017). Hence, it seems that the continued dependence on food sharing in wolves and the reduced reliance on this phenomenon in dogs may have affected some aspects of how individuals negotiate when and with whom to share.

In the current study, we investigated this aspect further by taking into account the quality of the social bond between individuals, both in terms of affiliation and rank. Rank and affiliation quality were determined on the basis of daily observational data of the animals interacting with their pack members (for ethogram, see supplementary Table S1). The effects of these factors on food sharing were assessed in two different contexts: a controlled dyadic *tolerance test* where two animals were simultaneously released onto a food source and a *carcass test* where the whole pack was provided with a single carcass. The former test allowed for a measure of each dyad’s behaviors without the potential interference of other pack members, whereas the latter allowed for measures of more naturalistic behaviors over a longer feeding period in a group environment. Additionally, the group tests allowed us to investigate the effect of carcass positioning on feeding behavior as we presented the carcasses suspended from a tree (hanging) or lying on the ground. The aim of the hanging carcass was to increase the effort required to access the food, simulating a more similar setting to “bringing down prey” in order to assess whether this may promote more cooperative feeding behavior, or at least reduce the opportunity for agonistic interactions.

Our primary prediction was that, because social relationships are important in both species (Harrington and Mech 1982; Bonanni et al. 2010), dyads with a stronger social bond should show more tolerance around food. In addition, we expected that in dogs at least, a higher difference in rank would promote more tolerance around the food than those dyads close in rank (Cafazzo et al. 2010). Furthermore, based on previous results (Range et al. 2015), we expected that dominance would strongly mediate feeding behavior in dogs but have a reduced or no effect in wolves. Specifically, we predicted that in dogs, but not wolves, dominant individuals would monopolize the food more, show more aggression, and show less peaceful sharing than subordinate animals. Furthermore, we predicted that having to pull down the carcass (i.e., when it was hanging) would increase tolerance and decrease aggressive interactions in wolves, which heavily rely on group hunting, but have a smaller or no effect on dogs (that mostly rely on scavenging).

Persistence in solicitation for food affects food sharing behavior and appears to be mediated by the strength of the social bond at least in chimpanzees (Eppley et al. 2013). Furthermore, in both wolves and dogs, persistence has been shown to vary based on female reproductive stage (Dale et al. 2017). Therefore, we expected individuals of both species to show more perseverance to access the food from those with whom they have a stronger affiliative bond (Eppley et al. 2013). However, due to the strong influence of rank on food tolerance in dogs, we also predicted that in this species, but not in wolves, subordinate individuals would show little persistence but rather maintain their distance from the food resource.

## Methods

### Subjects

Wolf and dog packs at the Wolf Science Center (www.wolfscience.at) were tested. The wolves originate from wild parks in America and Canada. The dogs are mixed breeds and most originate from shelters in Hungary. The eight dogs of the latest generation, which were present for the carcass feeding tests (but not the tolerance tests), were bred at the Wolf Science Center from Layla/Nia and external, mixed breed males. All subjects were hand-raised in peer groups from the age of 10 days. They were bottle-fed and later hand-fed by humans and had continuous access to humans as social partners in the first 5 months of their life. After 5 months, they were introduced into the packs of adult animals and currently live in large 2000–8000 m^2^ enclosures in these groups (see Range et al. 2015 for more details). As adults, they take part in training and various behavioral experiments on a daily basis, mostly using commercially available dry food and “extra wurst” sausage as rewards. Since divergence, dogs have evolved different digestive systems and feeding routines than wolves (Axelsson et al. 2013). In order to account for this and to create similar levels of food motivation in both groups, dogs are fed daily using scattered feeding and wolves are fed every 2–5 days with individual pieces of meat (e.g., rabbits). Both species had the same amount of experience with feeding tests and monopolizable food sources prior to this study and carcass feedings are used in public demonstrations on a weekly basis (one pack per week).

The current experiment tested nine dogs (5F, 4M) and 12 wolves (4F, 8M, see Tables 1 and S1 for details). Two methods were used to investigate food sharing responses, *dyadic food tolerance tests* and *carcass feedings* in the pack environment.

**Table 1:** Pack details for the naturalistic tests and number of carcass feedings presented to each

Pack	Species	# of individuals	Individuals	Hanging	Lying
Kaspar	Wolf	3M, 2F	Kaspar, Aragorn, Shima, Tala, Chitto	3	3
Geronimo_1	Wolf	3M	Geronimo, Amarok, Kenai	1	5
Geronimo_2	Wolf	2M, 1F	Geronimo, Wamblee, Yukon	3	3
Meru_1	Dog	4M, 2F	Meru, Nia, Gombo, Hiari, Sahibu, Imara	1	1
Meru_2	Dog	2M, 1F	Meru, Hiari, Imara	2	1
Nuru_1	Dog	4M, 3F	Nuru, Layla, Zuri, Pepeo, Enzi, Panya, Banzai	1	1
Nuru_2	Dog	3M, 3F	Nuru, Layla, Zuri, Pepeo, Enzi, Panya	1	2
Asali	Dog	2M, 1F	Asali, Bora, Banzai	2	4

## Food sharing tests

### Dyadic tolerance tests

Dyads always comprised two individuals from the same pack. The two animals were released onto a food source at the same time and tolerance around the food source was measured. Every dyadic combination from each pack completed three trials.

#### General set-up

Subjects were placed into separate, but adjacent, side compartments where they could see, but not enter, the central enclosure (see supplementary movie 1). A sliding door connected each compartment to the central enclosure. The animals were already accustomed to being temporarily separated and to moving through the doors when opened. The experimenter then walked into the central enclosure and visibly placed a shallow plastic bowl baited with 10 meat chunks and a handful of dry dog food (20 cm in diameter for dogs and 40 cm for wolves due to their different head sizes) in front of the animals, centrally between them at a distance of 3 m from each door and then left again. The bowl sizes were chosen to replicate Range et al. (2015) and accounted for the different head sizes and body weights of the animals (mean weight: dogs = 24.57 kg, wolves = 40.65 kg, mean head size: dogs = 40.46 cm, wolves = 52.15 cm), such that the bowls were large enough to allow the animals to eat from the same bowl simultaneously, but were also small enough so that an animal could easily monopolize it. The meat chunks are a highly desirable food for both the wolves and dogs and the dry food increased the total volume to allow each trial to last longer. The experimenter filmed all trials from the other side of the fence, at a distance of 5 m from the food location. For all trials, subjects were filmed until the food was finished or for a maximum of 2 min.

#### Individual trials

At the start of each session, each animal was individually released into the central enclosure through a sliding door and allowed to eat a handful of meat and dry food from the bowl before the test began. This was in order to make the animals aware that food rewards would be placed in the bowl and to ensure food motivation. Each subject received one individual trial before testing and the trial was filmed. If an animal was not motivated to eat in the individual trial, the test was not continued that day. Although the second individual could watch the individual trial, the order of the individual trials between the two subjects was randomized across sessions. Therefore, it was not always the same individual eating first. The handful of food in the individual trial was a small amount relative to the animal’s daily food intake; as such, it was highly unlikely that it affected their satiation levels enough to reduce motivation to access a large bowl of meat in the following test trial.

#### Test trials

Immediately after the individual trials, the subjects received one test trial where, after the experimenter placed the baited bowl in the central enclosure, the animals were simultaneously released into the enclosure through the sliding doors (see supplementary movie 1). The test ended when all the food was consumed or after 2 min.

### Carcass feedings

For the carcass feedings, the pack was presented with a deer carcass in their home enclosure. The wolves received an entire carcass and the dogs received a hind leg of a carcass, the difference in size being due to the differing daily feeding requirements of the two species. The animals were removed from their home enclosure, a procedure that is normal in their daily routine, and the carcass was chained to a tree within the enclosure. The carcass was chained to a tree either lying flat on the ground or suspended off the ground but low enough for the animals to reach (approximately 50 cm). The person then left and the animals were released back into the enclosure (supplementary movie 2). The carcass, and 10 body lengths surrounding it, was filmed from outside the enclosure through the fence at a distance of approximately 5 m, although this varied slightly depending on the enclosure. Each session commenced when the first animal approached within 10 body lengths of the carcass and ended after exactly 40 min.

## Analyses

### i. Characterization of social relationships

Regular focal observations of social interactions between pack members for this study have been conducted at the Wolf Science Center since June 2013. Ten-minute long focal animal samplings (Altmann 1974) were carried out for each individual using the Pocket Observer program (3.2 Software) and were then imported into the Observer XT 10.5 program (both from Noldus Information Technology, Wageningen, The Netherlands). Each individual’s observations were equally distributed as much as possible, over the time period, as well as across time of day. During focal sessions, agonistic and affiliative behaviors were recorded by the “all occurrences method” (Altmann 1974). The descriptions of all behavioral patterns used to determine social relationships can be found in the ethogram (supplementary materials Table S1). From these observations, the hierarchical structure of each pack, as well as the affiliative and dominance relationship of each dyad within a pack were ascertained (see below). The data is analyzed once per year as this provides sufficient data to calculate robust social relationship scores, and therefore, the relationship scores for each test were taken from the corresponding year.

### Dominance

#### Pack hierarchy

The dominance relationships in the packs were analyzed based on either the submissive or dominance behaviors exhibited depending on which provided the strongest linearity index (Cafazzo et al. 2010). Based on de Vries’ improved Landau’s linearity index (de Vries 1995), we found that each pack showed a linear hierarchy (i.e., a linearity index of over 0.75). The statistical significance of h′ was tested by means of a two-step randomization test with 10,000 randomizations (de Vries 1995) using MatMan 1.1 (Noldus Information Technology, Wageningen, The Netherlands). Pack members were therefore ordered using a procedure proposed by de Vries for finding a dominance order most consistent with a linear hierarchy (the I&SI (inconsistencies and sum of inconsistencies) method; de Vries 1998-MatMan program). This provided the values for the ordinal rank measure (with 1 being the most dominant) used in the carcass test analyses (see below).

#### Rank distance

In addition to the general hierarchy order of each pack, we were also interested in the specific dominance relationship between each pair of individuals in a pack. This relationship could be compared with the feeding behavior seen in the dyadic tolerance tests, when only those two individuals were present around the food source. In order to characterize the dyadic dominance relationship, we calculated the David’s score (Gammell et al. 2003) for each individual and then subtracted individual A’s score from individual B’s in order to obtain a value of rank distance for each pair. The advantage of using the David’s score for this measure is that it takes into account the relative strengths of other individuals in the group when calculating the relative dominance score of each animal.

### Affiliation

To characterize the quality of the relationship between individuals, an “affiliation score” was used (Silk et al. 2013). This represents the bidirectional frequency of affiliative behaviors (see observation ethogram) exchanged by individuals A and B, divided by observation time (h) for subjects A + B.

$$ \mathrm{Affiliation}=\frac{{\mathrm{A}}_{\mathrm{i}}+{\mathrm{B}}_{\mathrm{i}}}{{\mathrm{A}}_{\mathrm{j}}+{\mathrm{B}}_{\mathrm{j}}} $$

where i represents the frequency of affiliative behaviors and j represents the total number of observation hours.

### ii. Feeding tests

## Coding and inter-observer analyses

For both types of test, the videos of each session were coded with Solomon Coder Beta 15.01.13 (Copyright András Péter, http://solomoncoder.com). The ethograms used for the coding of feeding behavior can be found in the supplementary materials (Tables S2 and S3). In the tolerance tests, the behavior of both individuals in the dyad was coded, and in the carcass tests, the behavior of every individual in the pack was coded, as well as interactions with all other partners.

It was not possible to code the data blind because our study involved focal animals requiring individual recognition. The coding of the food tolerance tests was carried out by RD with 20% coded by LS for reliability (who was blind to the social relationships and hypotheses at this stage). Cohen’s kappa coefficient revealed a high level of agreement on all binomial variables, considering the guidelines by Landis and Koch (1977: >0.75 as excellent and 0.4–0.75 as good; aggression: 1.0, peaceful sharing: 0.91, feeding alone: 0.68). The carcass feedings were coded by LS and RD with 20% coded by both for reliability (food monopolization: 0.96, peaceful sharing: 0.91, aggression: 0.71, waiting: 0.91, begging: 0.78, arrive first: 1, close proximity: 0.77, far proximity: 0.87, defending: 0.28, scrounging: 0.39 (after consultation, these two variables had complete agreement)).

## Statistical analyses

To address our main questions, i.e., the influence of affiliative and dominance relationships on the food-sharing behavior of wolves and dogs, we carried out a series of models, with the above factors as explanatory variables and either the occurrence, frequency, or duration of specific behaviors relating to food sharing as our dependant variable.

Wolves and dogs could not be directly compared due to an imbalance in the number and sex composition of the dyads and packs (Tables 1 and S1). Nevertheless, the same models were run for both species. This method of analysis allowed for the investigation of the relevant social factors affecting feeding behavior in the two canid species.

## Tolerance tests

In the tolerance tests, we analyzed whether the duration of aggression, peaceful sharing (i.e., co-feeding from the same bowl with no signs of threat), and food monopolization (i.e., one individual feeding alone from the bowl; see supplementary materials for detailed definitions) were affected by the rank distance and affiliative relationship between the individuals in a dyad. Accordingly, we ran generalized linear mixed models (glmer function in the lme4 package) with a Gaussian distribution and logit link function. Our dependant variables were the durations of the behaviors/behavioral categories described above (divided by trial length), and the explanatory variables were the affiliation score and rank distance as measures of the quality of the relationship of each dyad. As some subjects were tested in multiple dyads and each dyad was tested repeatedly, we created a variable whereby each individual-dyad combination was given a unique identifier, and this was inserted as the random effect in the models.

In addition, due to a higher variation in the likelihood of peaceful co-feeding occurring at all in the wolves (see results), we also ran a GLMM with a binomial distribution to assess whether the likelihood of occurrence of peaceful co-feeding was affected by the social relationship. The same fixed and random effects were included in the model.

For all GLMMs, model assumptions were met. The following construct depicts the model used for all GLMMs for the tolerance tests:

$$ {\mathrm{Response}}_{\mathrm{i}\mathrm{j}\mathrm{k}}\sim {\mathrm{affiliation}}_{\mathrm{i}}+\operatorname{rank}\_{\mathrm{distance}}_{\mathrm{j}}+{\mathrm{animal}}_{\mathrm{i}\mathrm{j}}+{\mathrm{e}}_{\mathrm{i}\mathrm{j}\mathrm{k}} $$

In this model, affiliation_i_ is the fixed effect of a dyad’s affiliation score and rank_distance_j_ is the fixed effect of the rank distance between two individuals of a dyad. Animal_ij_ is the random animal within dyad effect with mean zero and e_ijk_ is the random residual with mean zero.

## Carcass tests

As with the dyadic tests, dogs and wolves were analyzed separately. In the carcass tests, two types of behaviors were coded: (1) behaviors that had a recipient (i.e., peaceful sharing, aggression, waiting, begging, and defending) and (2) behaviors that did not have a recipient (food monopolization, scrounging, arriving first at the carcass, proximity to the carcass) (see supplementary ethogram for detailed descriptions of each behavior). All behaviors that occurred within 10 body lengths of the carcass were coded.

As in the tolerance tests, for behaviors that had a recipient, the fixed effects for all models were affiliation score and ordinal rank. Carcass position (hanging or lying) was also included as a fixed effect for the peaceful sharing and aggression models. Ordinal rank was used rather than rank distance between two individuals (as for the dyadic testing) since our main question in this group context was whether the behaviors exhibited during feeding were affected by the individuals’ position in the pack hierarchy. A variable whereby each individual-dyad-pack combination was given a different name was included as the random effect.

For the behaviors that did not have a recipient, we assessed the impact of hierarchical position in the pack on the occurrence/duration of these variables. For these variables, a random factor with each individual-pack combination was created and ordinal rank was the fixed effect.

For all behaviors, if its occurrence was relatively frequent (i.e., occurred in more than 50% of data points) and met the model assumptions, either the frequency (aggression, carcass proximity) or the duration (peaceful feeding, food monopolization) was entered in the model as the dependant variable, whereas if the behavior was infrequent, analyses were carried out on the likelihood of its occurrence (waiting, begging, defending, scrounging, arriving first). Accordingly, GLMMs with a Gaussian distribution (package: lmer) were run for duration variables, glmmPQL for frequencies (package: nlme), and GLMM with a binomial distribution for 1/0 variables (package: lmer).

All analyses were carried out in R version 3.2.2 (R Core Team 2015).

## Results

### Tolerance tests

**Fig. 1:**
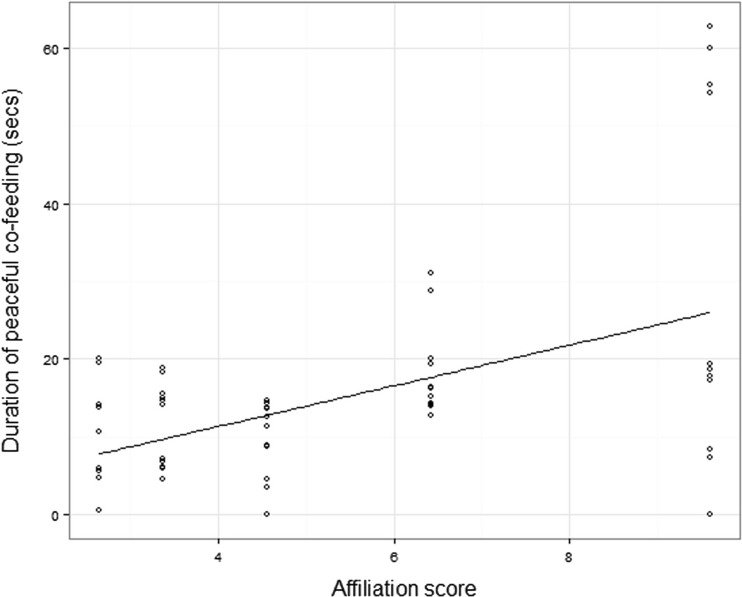
The higher the affiliation score of a dog dyad, the longer they peacefully co-fed for in the tolerance tests

In both wolves and dogs, peaceful sharing was mediated by the social relationship between individuals. In dogs, peaceful co-feeding occurred on 97% of trials, but how long it lasted for was strongly affected by the relationship with the partner; the higher the affiliation score, the more time dyads spent peacefully sharing (Fig. 1), but no effect of rank distance emerged. In wolves, peaceful co-feeding occurred on 83% of trials, and here, the duration was not affected by affiliation or rank distance. However, due to this higher variation in whether or not sharing occurred, we also analyzed the likelihood of peaceful sharing occurring in a trial and this was higher in dyads with a higher affiliation score and a higher rank distance. Full model results are presented in Table 2.

**Table 2:** Full model results from the tolerance tests for each variable and species

	Wolves		Dogs	
	Affiliation	Rank distance	Affiliation	Rank distance
	Duration		Duration	
Peaceful sharing	χ^2^ = 0.75 (1), *p* = 0.39	χ^2^ = 1.25 (1), *p* = 0.26	χ^2^ = 7.14 (1), *p = 0.008*	χ^2^ = 2.05 (1), *p* = 0.15
	Likelihood			
Peaceful sharing	χ^2^ = 6.99 (1), *p = 0.008*	χ^2^ = 11.57 (1), *p < 0.001*		
	Duration		Duration	
Food monopolization	χ^2^ = 1.43 (1), *p* = 0.2	χ^2^ = 2.46 (1), *p* = 0.12	χ^2^ = 1.72 (1), *p* = 0.19	χ^2^ = 0.04 (1), *p* = 0.95
	Duration		Duration	
Aggression	χ^2^ = 0.22 (1), *p* = 0.64	χ^2^ = 0.31 (1), *p* = 0.58	χ^2^ = 1.35 (1), *p* = 0.25	χ^2^ = 0.97 (1), *p* = 0.32

Neither the affiliation score nor the rank distance of a dyad affected the durations of aggression or food monopolization shown in either species (Table 2).

### Carcass tests

**Fig. 2:**
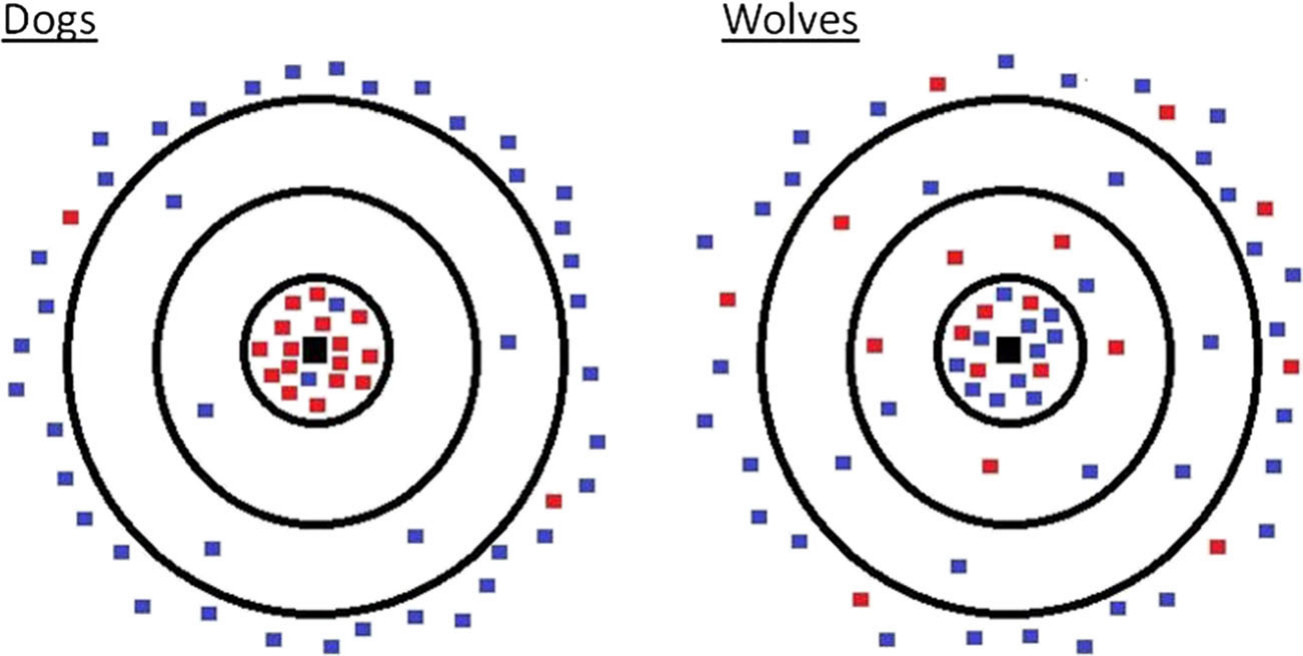
A typical picture of how a carcass feeding session looks in dogs (*left*) and wolves (*right*). *Red dots* represent the most dominant individual and *blue dots* all other pack members. Each dot is where one individual spent most of their time (the greatest number of proximity scans during the session) in one session. The rings denote distance from the carcass: 0–1, 1–5, and 5–10 body lengths

The typical pattern of a carcass feeding was generally the same across sessions and packs but varied according to the species tested (see supplementary movie 2 and Fig. 2 for examples of how the feedings looked in each species). The full model outputs for the carcass tests are presented in Tables 3, 4, and 5.

**Table 3:** Results of the duration of peaceful sharing, food monopolization, and the frequency of aggression in the carcass feedings dependent on affiliation score (continuous score based on observations), rank (linear position), or carcass position (hanging vs lying)

		Wolves			Dogs		
Variable	Fixed effect	χ^2^	*df*	*p* value	χ^2^	*df*	*p* value
Peaceful sharing (duration)	Affiliation score	2.27	1	0.13	0.03	1	0.64
	Ordinal rank	0.0.8	1	0.77	*17.08*	*1*	*0.0001*
	Carcass position	0.03	1	0.86	2.29	1	0.13
		Wolves		Dogs			
	Fixed effect	χ^2^	*df*	*p* value	χ^2^	*df*	*p* value
Aggression (frequency)	Affiliation score	0.55	1	0.46	2.00	1	0.16
	Ordinal rank	*7.00*	*1*	*0.008*	*30.77*	*1*	*0.0001*
	Carcass position	*6.12*	*1*	*0.01*	0.25	1	0.61
		Wolves			Dogs		
	Fixed effect	χ^2^	*df*	*p* value	χ^2^	*df*	*p* value
Food monopolization	Ordinal rank	0.36	1	0.55	*5.45*	*1*	*0.02*

**Table 4:** Model outputs for the effects of ordinal rank (and affiliation score) on the following variables

Variable	Fixed effect	Wolves			Dogs		
*Frequency*		χ^2^	*df*	*p* value	χ^2^	*df*	*p* value
#scans spent <1 body length from carcass	Ordinal rank	0.46	1	0.50	*6.73*	*1*	*0.009*
#scans spent >10 body lengths from carcass	Ordinal rank	2.41	1	0.12	*5.33*	*1*	*0.02*
*Binomial*		χ^2^	*df*	*p* value	χ^2^	*df*	*p* value
Waiting	Ordinal rank	1.48	1	0.22	0.07	1	0.79
	Affiliation score	0.44	1	0.51	0.1	1	0.76
Begging	Ordinal rank	0.04	1	0.84	0.15	1	0.70
	Affiliation score	0.92	1	0.34	0.69	1	0.41
Defending	Ordinal rank	*3.64*	*1*	*0.05*	*4.60*	*1*	*0.03*
	Affiliation score	0.15	1	0.69	1.27	1	0.26
Scrounging	Ordinal rank	0.18	1	0.67	0.03	1	0.86
Arrive first	Ordinal rank	*4.54*	*1*	*0.03*	2.52	1	0.11

**Table 5:** Results from the glmms comparing subordinate wolves and dogs on the likelihood of the occurrence of the following variables

*Binomial*	Fixed effect	χ^2^	*df*	*p* value
Scrounging	Species	*5.85*	*1*	*0.01*
Waiting	Species	*3.68*	*1*	*0.05*
Begging	Species	0.001	1	0.97

More dominant wolves were more likely than lower ranking wolves to defend the carcass, preventing others from approaching despite not eating themselves, and were more likely to arrive first at the carcass at the start of the session, but were not more likely to monopolize the carcass for feeding than lower ranking animals. Moreover, rank did not affect how much time wolves spent close (<1 body length) or far (>10 body lengths) from the carcass (Fig. 2).

**Fig. 3:**
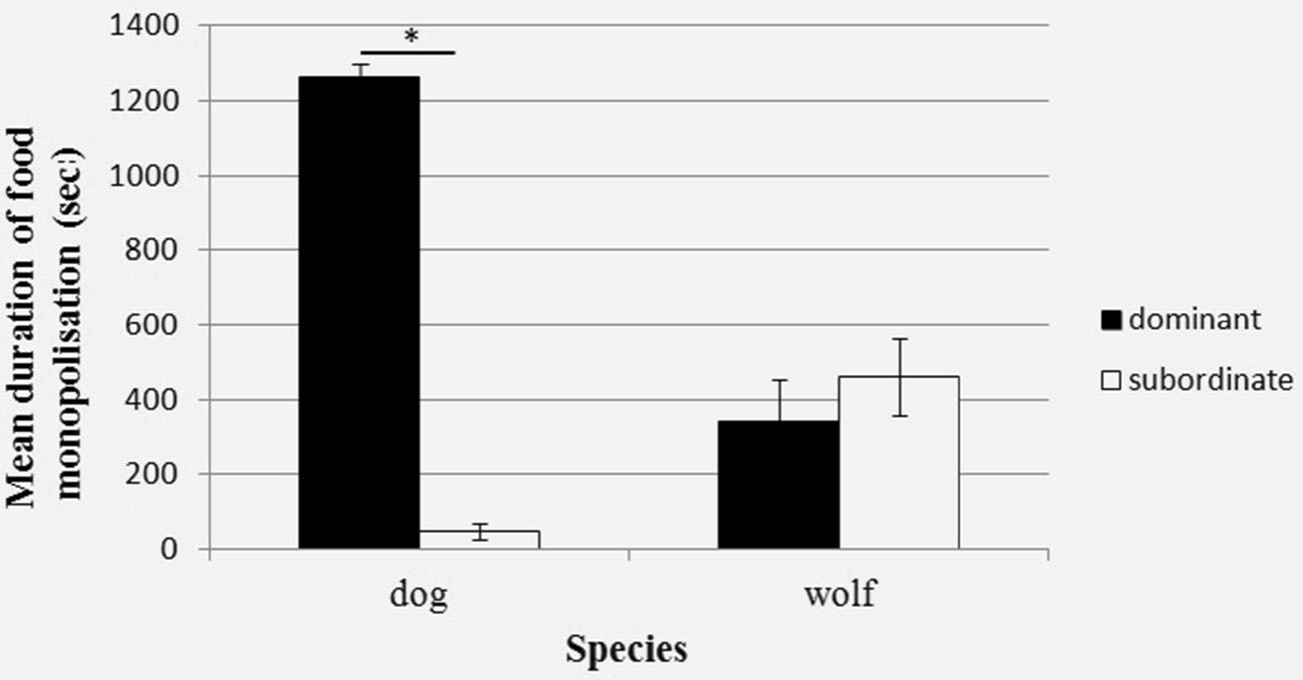
Mean duration of food monopolization (sec) by the most dominant member vs all other pack members. * < 0.05. *Error bars* represent the standard error of the mean

On the contrary, the dominant dogs, although not more likely to arrive first, spent significantly more time monopolizing the carcass (specifically the most dominant dog; Fig. 3) and were more likely to defend it from others than subordinate dogs. In fact, in 40 min of carcass test, subordinate dogs (i.e., all but the most dominant individual) spent an average of less than 50 s feeding alone at the carcass (almost 10 times less than subordinate wolves, mean = 462 s). Furthermore, proximity to the carcass was significantly affected by rank in the dogs. More dominant dogs were significantly more likely to spend time close to the carcass than subdominants, whereas subordinate dogs spent more time at a great distance from the resource (>10 body lengths; Fig. 2). In fact, as seen in Fig. 2, this was not a gradual effect of rank, with distance increasing as rank position decreased, but rather that the most dominant individual monopolized the proximate area around the carcass and all other individuals mostly stayed away.

The duration of peaceful sharing in wolves was not affected by affiliation score, ordinal rank position, or carcass position (Table 3). However, aggression was affected by ordinal rank and carcass position. Higher ranked individuals showed more aggression than subordinates, but interestingly, aggression was less frequent when the carcass was hanging than when it was lying. Affiliation score, however, did not affect the amount of aggression shown towards a particular partner (Table 3).

In the dogs, the duration of peaceful sharing and frequency of aggression were strongly affected by rank position, with higher ranking dogs spending less time peacefully co-feeding than lower ranked individuals and mainly the most dominant individual showing aggression. Neither the duration of peaceful sharing nor the frequency of aggression were affected by carcass position (hanging vs lying) or affiliation score in the dogs (Table 3).

Begging and waiting (coded as measures of persistence) were not affected by ordinal rank or affiliation score, nor was the likelihood of scrounging affected by rank position in either species (Table 4).

From the results above, it appears that in the dogs, the subordinate animals were staying away from the carcass altogether (Fig. 2). However, the wolves did not show such an effect of rank on proximity to the carcass. Therefore, we were interested in what it was that the subordinate wolves were doing that the dogs were not. For only the subordinate animals (here considered as every individual other than the most dominant pack member), we compared the wolves and dogs in scrounging, begging, and waiting. There was no effect of species on the likelihood of begging but subordinate wolves were significantly more likely to scrounge and wait than subordinate dogs (Table 5).

## Discussion

In sum, results from the dyadic tolerance tests indicate that in both species, the affiliative relationship with a partner is the most relevant factor in dictating the level of tolerance shown around the food source, with more peaceful sharing occurring in dyads with higher affiliation scores. Unsurprisingly, the picture is a little more complex in the carcass tests, where the whole pack was present. However, the primary difference with the dyadic tolerance tests was that affiliation did not emerge as predictive of food tolerance in this context. Rank, on the other hand, was the more important factor in determining an individual’s behavior around the food in the group setting. For wolves, but not for dogs, the hanging carcass also affected their behavior by reducing the frequency of aggressive interactions.

The results from the dyadic tolerance tests support our primary prediction, demonstrating that when just two individuals are presented with a situation of a small food source, the stronger their affiliative bond the more they engaged in peaceful sharing. It is logical that animals should choose to co-feed with partners who are most likely to show tolerance, than a less close affiliate, who may challenge you or incite conflict (de Waal 2000; Heesen et al. 2014). Interestingly though, how this effect of affiliation emerged appears to differ between the two species. In the dogs, co-feeding occurred on 97% of trials, but the closeness of the social bond affected the time spent co-feeding. In the wolves, no such effect was seen on the duration of peaceful sharing, but it seems that their decision about whether or not to share at all was based on the affiliative relationship, as those with a higher affiliation score were more likely to peacefully share. At this stage, we cannot conclude whether this difference is due to a species difference in how the animals decide whether/how to share or whether this is driven by the imbalance in sex compositions between the species, as we had mostly male-female pairs in the dogs but all possible sex compositions in the wolves.

In the wolves, we also found that those dyads with a higher rank distance between the individuals were more likely to peacefully share. This may be due to age, with young individuals often occupying low dominance ranks and/or it may be because those lower in rank present less competition to the status position/mating opportunities of high-ranked individuals. These findings are in line with those by Cafazzo et al. (2010) in free-ranging dogs that high ranking dogs often showed aggression to mid-ranking individuals but displayed a certain level of tolerance towards low-ranking individuals. However, interestingly, the results are in contrast to findings in primates, where those close in rank are predicted to feed together (Seyfarth 1977; Matsumura and Okamoto 1997; Tiddi et al. 2012), even after controlling for kinship (de Waal 1991; but see Kapsalis and Berman 1996). The fact that we did not find this effect in the dogs may reflect a species effect and/or is possibly because, differently from wolves, dog pairs were mostly male-female and factors other than rank distance may also play a role in their feeding behavior (Dale et al. 2017).

The affiliative relationship shared by individuals did not affect any of the coded behaviors around the food in the carcass context. This may be because in a group setting, you cannot necessarily co-feed with your “friends” as they may not be permitted by other pack members to access the resource. In this situation, it was the rank that predominantly affected the behavior of the animals. In the wolves, the individuals higher in the hierarchy showed more aggression, were more likely to arrive first at the carcass, and were more likely to defend it (i.e., not feeding, but preventing others from approaching) than pack members lower in rank. However, they were not more likely to spend time close to the carcass than more subordinate individuals nor did they monopolize the resource more than others, suggesting that the dominant wolves were trying to control access to the food, but not monopolizing it for themselves (Noë et al. 1980). Indeed, it has been found that in rooks, food offering acted as a costly signal of rank, whereas tolerated co-feeding was explained by reciprocity and formation of social bonds (Scheid et al. 2008). These findings corroborate with our results in wolves that affiliation was important in peaceful sharing but rank determined defense of the carcass. Furthermore, this interpretation corresponds with data from wild wolves which suggests that the dominant individuals choose whom to allot food to, and this in turn ensures the survival of the whole pack (Mech 1999).

Although rank was also an important factor in dog carcass feeding behavior, it was in a different manner to that of the wolves. Dominant dogs were, like the wolves, more likely to defend the carcass and showed more aggression than subordinate individuals. However, this appeared to be in order to maintain and feed on the carcass themselves, since they were also less likely to co-feed and much more likely to monopolize and spend time in close proximity to the resource than more subordinate individuals, despite being no more likely than subordinates to arrive first. These results support our second hypothesis that feeding behavior in dogs would be more dependent on rank than in wolves.

Furthermore, the results also corroborate those of Range et al. (2015), who found that in a dyadic context, feeding was mediated by rank in the dogs, but not in the wolves. Our results from the tolerance tests of the current study are not directly comparable with those of the previous research as we had mostly male-female dog pairs during this phase of the research. However, results from the carcass tests (where same sex relationships were included) support the interpretation that dogs show much less tolerance, and a steeper hierarchy around food sources than wolves with the most dominant dog mostly monopolizing the resource and the rest of the pack spending most of the session more than 10 body lengths away from the carcass. So, even though the results refer to the animals’ ordinal rank position in the pack, a closer look at the raw data (highlighted in Figs. 2 and 3) shows that it is only the most dominant dog that monopolizes the food source. In contrast, the wolves did not show an effect of rank on proximity to the carcass during a session, meaning that both dominant and subordinate animals were as likely to be close to it. Dubuc and Chapais (2007) suggest that an individual’s spatial position during group feeding affects potential feeding gains, but the position of subordinates may be mediated by the tolerance of the dominants (see also King et al. 2011). In line with this, our results suggest that subordinate wolves, but not dogs, are tolerated close to the carcass and are more likely to gain access to it.

It is clear that subordinate dogs (here considered all but the most dominant individual) tended to avoid the carcass altogether, but in order to ascertain what it was that subordinate wolves were doing, we compared the subordinate individuals of both species. We found that wolves were more likely to show persistence behaviors (namely waiting and scrounging) than the dogs were. Eppley et al. (2013) suggested that persistence is not a strategy available to all individuals; tolerance is required by a food possessor before an individual will demonstrate persistence behaviors. Therefore, subjects tend to “beg” from possessors with whom they have a strong social bond. We had predicted that this would also be the case in the current study, but here, the restricted use of persistence seems to be at play at the species level instead, with wolves being more able to make use of persistence as a strategy to access the food than dogs. Subordinate wolves also had as much success as dominant wolves in monopolizing the carcass, further suggesting that rank is less strictly enforced in the feeding context in wolves. These findings support our predictions of higher levels of persistence from subordinate wolves and the avoidance of the resource by the subordinate dogs.

Another strategy suggested as a tactic for subordinates to gain access to food is arriving first at the resource (Dubuc and Chapais 2007). Dubuc and Chapais (2007) found that in long-tailed macaques, rank did not determine the order of arrival at a feeding site, but early arrival did allow for more food consumption. Interestingly, our results appear to contradict this as we found that dominant wolves were more likely to arrive first, but did not monopolize the food more than subordinates. In contrast, subordinate dogs were as likely to arrive first at the carcass but nevertheless were unable to monopolize the food. This suggests a potential difference in the use of first arrival, with our animals appearing not to use this as a tactic for resource access.

Another factor that we considered was how the position of the carcass may affect the tolerance of these two species. Partially supporting our final prediction, wolves (but not dogs) showed less aggression when the carcass was suspended from a tree than when it was lying on the ground, but the position did not affect the duration of peaceful sharing. This potentially suggests that a hanging carcass promotes cooperation rather than competition, as would be required to bring down prey in wild situations and in turn, the process of bringing down prey may be a factor that helps maintain low aggression levels in wild wolves. Alternatively, when the carcass is hanging, the wolves may be so distracted by the desire to pull it down, that they have less focus on the other individuals around. Regardless of the underlying motive, this finding potentially affects the management of captive wolf populations, suggesting that feedings could include this feature as it may promote more tolerance. From an evolutionary perspective, the fact that it did not affect the dogs may be because, although they cooperate with humans, they are no longer as cooperative with conspecifics (Range and Viranyi 2015; Marshall-Pescini et al. 2017).

It could be argued that the different feeding routines and bowl and carcass sizes used with the wolves and dogs affected the results. However, the feeding routines are different in order to match the requirements of each group. Wolves and dogs show differences in feeding ecologies and digestive systems; wolves have a meat-based diet and typically eat every few days since they are required to hunt for their meals, whereas dogs scavenge on a daily basis and eat a more starch-rich diet (Vanak and Gompper 2009b; Axelsson et al. 2013). Additionally, the bowl and carcass sizes were chosen according to the body sizes of the animals, such that in both species, the resource could be shared, but was small enough to be monopolized if an individual so desired. Furthermore, in neither species does the daily feeding involve restricted, monopolizable resources, and both groups had the same amount of experience with feeding tests prior to this study. However, although we feel that these factors do not prevent a comparison between wolves and dogs, we are aware that these factors are different from the feeding situations of free-ranging individuals. It is interesting to note that despite the limitations of testing and generalizing from captive populations, the results from both species do align with their respective social organizations and feeding ecologies in free-ranging settings (Marshall-Pescini et al. 2017). Because wolves are cooperative hunters (MacNulty et al. 2012), it is essential for the survival of the pack that every member is able to access food resources, regardless of their rank. Dogs, on the other hand, rely predominantly on solitary scavenging from human waste (Butler et al. 2004; Vanak and Gompper 2009a); therefore, it is not necessary to allow other group members to access a resource, and in fact, this may even harm your own fitness. These differing feeding ecologies appear to be reflected in our results from the carcass tests, whereby subordinate wolves are able to exhibit more persistence and feed from the carcass considerably more than subordinate dogs.

Overall, what emerges from these tests is that the social relationship with a partner affects an individual’s behavior around a food source in both wolves and dogs. However, this effect is mediated by context, with the affiliative relationship being the driving predictor of tolerance in a restricted, dyadic setting but rank overiding this in the group tests, where the whole pack is present. Furthermore, the wolves’ reliance on cooperation allows all pack members access to the food, whereas dogs show more despotic behavior around food, with the dominant individual monopolizing the carcass. These tests reveal that social factors are also important in determining the feeding behavior of canids and highlight the benefits of using multiple contexts and species in order to ascertain the socio-ecological factors driving the distribution of food in social groups.

## Electronic supplementary material

ESM 1 (DOCX 22 kb).

ESM 2 (DOCX 14 kb).

ESM 3 (AVI 6713 kb).

ESM 4 (WMV 112641 kb).

ESM 5 (XLSX 62 kb).

ESM 6 (XLSX 100 kb).
