# Supplementary material for: The influence of social relationship on food tolerance in wolves and dogs
Source: Behav Ecol Sociobiol. 2017 Jun 30;71(7):107. doi: 10.1007/s00265-017-2339-8 (PMC5493712; doi:10.1007/s00265-017-2339-8)
Supplement: Supplementary file 1 — (DOCX 22 kb). [file 265_2017_2339_MOESM1_ESM.docx]

**The influence of social relationship on food tolerance in wolves and dogs.**

**Behavioral Ecology and Sociobiology**

**Authors:** Rachel Dale, Friederike Range, Laura Stott, Kurt Kotrschal, Sarah Marshall-Pescini

**Corresponding author:**Rachel Dale
[rachel.dale@vetmeduni.ac.at](mailto:rachel.dale@vetmeduni.ac.at)

Comparative Cognition, Messerli Research Institute, University of Veterinary Medicine, Medical University of Vienna, University of Vienna, Vienna, Austria.

Wolf Science Center, Messerli Research Institute, University of Veterinary Medicine, Vienna, Austria.

Supplementary materials 1

**Table S1: Ethogram for focal observations to calculate affiliation and rank relationships between pack members.**

| **WSC (Wolf Science Center) Ethogram for Pocket Observer** | | | |
| --- | --- | --- | --- |
| Behaviors | Description |  |  |
| **AFFILIATIVE INTERACTIONS** | | | |
| **Grooming (gro)** | To nip, lick or scratch the fur or skin. | |  |
| *Modifier:* Animal ID |  | |  |
| **Inspection (in)** | To interact in a friendly manner, stand next to each other, rubbing against each other side by side, smelling each other, putting heads together and licking, sniffing and so on. | |  |
| *Modifier:* Animal ID |  | |  |
| **Play invitation (pi)** | *Play bow* (one subject crouches down touching or almost touching forelimbs to the ground with rear end high in the air, orientation is directed towards play partner); *Start-stop*: from a stand-still position, looking directly at play partner, the subject quickly lowers front end of body (though not as low as a play bow) and/or quickly bounces up in front of partner);  *Bounce-bow*: similar to the start-stop, but involves repeated bounces as the actor appears to ‘dance’ around the play partner. | |  |
| *Modifier:* Animal ID |  | |  |
| **Lie friendly (lf)** | To lie on the back, tail-wagging, maybe kicking with the foreleg against another subject sometimes with open mouth. | |  |
| *Modifier:* Animal ID |  | |  |
| **Stand friendly (sf)** | The subject stands with tail perpendicular to or below the plane of the back, wagging it, ears pointed forward, while another is approaching it or orienting/looking towards it. | |  |
| *Modifier:* Animal ID |  | |  |
| **Body contact (bc)** | Two subjects stay (for at least 10 s) with at least a part of their bodies in contact and in a relaxed position. | |  |
| *Modifier:* Animal ID,  Activity (rest, sleep, other) |  | |  |
| **Social sniff (sl)** | To sniff another’s body part except its anogenital area. | |  |
| *Modifier:* Animal ID |  | |  |
| **Body rubbing (br)** | To rub one’s body against any part of the receiver’s. | |  |
| *Modifier:* Animal ID |  | |  |
|  | **DOMINANCE BEHAVIORS** | |  |
| **Stand tall (st)** | Subject straightens up to full height, with a rigid posture and tail, may include raised hackles, ears erect and tail perpendicular or above the back. | |  |
| *Modifier:* Animal ID |  | |  |
| **Stand over (so)** | To stand over another's body, with all four paws on the ground. The receiver may have either the whole body or just the forepaws under the actors’ belly/side. With tail held high. | |  |
| *Modifier:* Animal ID |  | |  |
| **Paw on** | To place one or both forepaws on the other’s back. | |  |
| *Modifier:* Animal ID |  | |  |
| **Ride up (ru)** | To mount another one from behind or from the side, exhibiting a thrusting motion. | |  |
| *Modifier:* Animal ID |  | |  |
| **Head on (ho)** | The subject approaches another’s shoulder/back and puts its head on it. Most of times formation looks like a capital “T”. | |  |
| *Modifier:* Animal ID |  | |  |
| **Muzzle bite (mz)** | To grab the muzzle of another subject either softly or with enough pressure to make the other whimper. | |  |
| *Modifier:* Animal ID |  | |  |
|  | **SUBMISSIVE BEHAVIORS** | |  |
| **Crouch (cr)** | Lowering the head, sometimes bending the legs, arching the back, lowering the tail between the hind legs, and avoiding eye contact. | |  |
| *Modifier:* Animal ID, Submission cause |  | |  |
| **Passive submission (ps)** | To lie on the back showing the stomach and holding the tail between the legs. The ears are held back and close to the head, and the subject raises a hind leg for inguinal presentation. | |  |
| *Modifier:* Animal ID, Submission cause |  | |  |
| **Active submission (acs)** | The subject has its tail tucked between the hind legs sometimes wagging it while he is in a crouched position (with hindquarters lowered) and may attempt to paw and lick the side of actor’s/aggressor’s muzzle. The behavior may include urination. | |  |
| *Modifier:* Animal ID, Submission cause |  | |  |
| **Withdrawing (wd)** | It occurs when a subject has been threatened or attacked by another, or a fight has taken place. The subject withdraws from another moving away slowly in the opposite direction, displaying a submissive posture. | |  |
| *Modifier:* Animal ID, Submission cause |  | |  |
| **Flee (fl)** | To run away from another with tail tucked between the legs and body ducked. It occurs when a subject has been threatened or attacked by another, or after a fight. | |  |
| *Modifier:* Animal ID, Submission cause |  | |  |
| **Avoidance (av)** | In response to another reducing the distance towards it, the subject moves away displaying a submissive posture. The subject may also look at the individual he is trying to avoid. | |  |
| *Modifier:* Animal ID, Submission cause |  | |  |

**Table S2: Ethogram for coding the tolerance tests**

Feeding behaviors (continuous):

**Peaceful co-feeding:** both animals are feeding from the same bowl without any communication.

**Food monopolization**: the subject is feeding alone.

Aggressive Behaviors (continuous):
All behaviors below were grouped as ‘aggressive behavior’. All instances of aggression were coded.

**Pushing**: during co-feeding, one animal physically pushes the other with the muzzle or body

**Threat**: oriented to the other with any of the following: curling of the lips, bearing of the canines, raising the hackles, snarling, growling and/or barking.

**Snapping**: to snap teeth into the air noisily.

**Pin**: to grab the other at the neck or at the muzzle, forcing it down to the ground and holding it there.

**Charge**: walks or runs towards the other with piloerection, stiff forelegs and ears back

**Chase**: runs after the other, usually with ears back and piloerection

**Attack**: running or jumping approach towards the other with tail, ears and sometimes hackles up, often bites at neck or muzzle.

**Fight**: a high intensity aggressive and often damaging encounter

**Bite**: closing the jaws or teeth on the other

**Knock down**: striking the other sharply so that they fall to the ground

Affiliative behaviors (instant)-
All behaviors below were grouped as ‘affiliative behavior’. All instances of these behaviors were coded.

**Play paw:** an animal paws on another’s body part

**Play bow:** one subject crouches down touching or almost touching forelimbs to the ground with rear end high in the air, orientation is directed towards play partner

**Stand friendly**: The subject stands with tail perpendicular to or below the plane of the back, wagging it, ears pointed forward.

**Body rubbing**: to rub one’s body against any part of the receiver’s.

**Table S3: Ethogram for coding the carcass tests**

**Start** (instant): when the first animal approaches within 10 body lengths of the carcass. Marks the start of the session.

**Stop** (instant): after 40 minutes (2420 seconds exactly, final proximity at 2400 seconds). Marks the end of the session.

**Proximity to carcass**: Scan sampling every 2 minutes of the proximity of each individual to the carcass:

<1 body length, 1-5 lengths, 5-10 lengths, >10 lengths

The first interval is coded 120 seconds after “start” (zero point). Each subject must fit into one of these options for each interval. For a 45 minute video there should be 22 intervals per subject. If not visible assume >10 body lengths.

Feeding behaviors (continuous)

**Feeding alone**- individual is the only pack member feeding at the carcass. Feeding defined as nose to the ground/on the carcass within one body length of the carcass.

**Peaceful co-feeding**- Subject is feeding with one or more other individual(s) and is eating from the carcass silently. Feeding defined as nose to the ground/on the carcass within one body length of the carcass. Select other individual(s) present.

**Scrounging**- feeding on small pieces of food that are unattached to the main carcass, at any distance from the carcass. Feeding is considered when an animal has their nose to the ground.

**Waiting**- the individual waits (stationary or moving) and has their head turned towards the food with a neutral stance, but does not eat. Lasts at least 5 seconds. Select other individual(s) present at the carcass.

**Begging** - Individual does not feed and shows affiliative behavior towards the individual(s) at the carcass. Lasts at least 5 seconds. Select other individual(s) present at the carcass.

Aggressive Behaviors:

- **Threat**: oriented to the other with any of the following: curling of the lips, baring of the canines, raising the hackles, snarling, growling, barking.
- **Snapping**: to snap teeth into the air noisily.
- **Pin**: to grab the other at the neck or at the muzzle, forcing it down to the ground and holding it there.
- **Charge**: walks or runs towards the other with piloerection, stiff forelegs and ears back
- **Chase**: runs after the other, usually with ears back and piloerection.
- **Attack**: running or jumping approach towards the other with tail, ears and sometimes hackles up, often bites at neck or muzzle.
- **Fight**: a high intensity aggressive and often damaging encounter
- **Bite**: closing the jaws or teeth on the other
- **Knock down**: striking the other sharply so that they fall to the ground

Defending

An individual is *not eating and is any distance from the carcass*, but prevents others from approaching the carcass by:

- - threatens or chases another that attempts to approach the carcass
  - moves in the direction of the carcass or stands tall while another attempt to approach.
