# Supplementary material for: The influence of social relationship on food tolerance in wolves and dogs
Source: Behav Ecol Sociobiol. 2017 Jun 30;71(7):107. doi: 10.1007/s00265-017-2339-8 (PMC5493712; doi:10.1007/s00265-017-2339-8)
Supplement: Supplementary file 2 — (DOCX 14 kb). [file 265_2017_2339_MOESM2_ESM.docx]

**The influence of social relationship on food tolerance in wolves and dogs.**

**Behavioral Ecology and Sociobiology**

**Authors:** Rachel Dale, Friederike Range, Laura Stott, Kurt Kotrschal, Sarah Marshall-Pescini

**Corresponding author:**Rachel Dale
[rachel.dale@vetmeduni.ac.at](mailto:rachel.dale@vetmeduni.ac.at)

Comparative Cognition, Messerli Research Institute, University of Veterinary Medicine, Medical University of Vienna, University of Vienna, Vienna, Austria.

Wolf Science Center, Messerli Research Institute, University of Veterinary Medicine, Vienna, Austria.

Table S4: The social relationship data for each dyad tested in the tolerance tests. All possible dyads were tested.

| Dyad | Species | Sex combination | *Affiliation score* | *Rank distance* |
| --- | --- | --- | --- | --- |
| *Kaspar-Aragorn* | *wolf* | *M - M* | 2.03 | 4.65 |
| *Kaspar-Tala* | *wolf* | *M - F* | 2.07 | 6.19 |
| *Kaspar-Shima* | *wolf* | *M - F* | 2.08 | 14.11 |
| *Kaspar-Chitto* | *wolf* | *M - M* | 1.32 | 12.71 |
| *Aragorn-Tala* | *wolf* | *M - F* | 1.83 | 1.54 |
| *Aragorn-Shima* | *wolf* | *M - F* | 1.5 | 9.46 |
| *Aragorn-Chitto* | *wolf* | *M - M* | 2.11 | 8.06 |
| *Tala-Shima* | *wolf* | *F -F* | 0.69 | 7.92 |
| *Tala-Chitto* | *wolf* | *M - F* | 4.05 | 7.92 |
| *Shima-Chitto* | *wolf* | *M - F* | 1.77 | 1.4 |
| *Nanuk-Yukon* | *wolf* | *M - F* | 0.67 | 2.77 |
| *Nanuk-Una* | *wolf* | *M - F* | 1.83 | 2.95 |
| *Yukon-Una* | *wolf* | *F -F* | 2.85 | 0.18 |
| *Geronimo-Amarok* | *wolf* | *M - M* | 2.05 | 3.0 |
| *Geronimo-Kenai* | *wolf* | *M - M* | 0.87 | 4.0 |
| *Amarok-Kenai* | *wolf* | *M - M* | 1.35 | 1.0 |
| *Geronimo-Yukon* | *wolf* | *M - F* | 0.52 | 3.0 |
| *Geronimo-Wamblee* | *wolf* | *M - M* | 0.59 | 6.0 |
| *Yukon-Wamblee* | *wolf* | *M - F* | 0.91 | 3.0 |
|  |  |  |  |  |
| *Maisha-Binti* | *dog* | *M - F* | 1.65 | 1.33 |
| *Asali-Bora* | *dog* | *M - F* | 3.37 | 1.75 |
| *Meru-Nia* | *dog* | *M - F* | 9.59 | 0.88 |
| *Nuru-Layla* | *dog* | *M - F* | 2.65 | 0.59 |
| *Nuru-Zuri* | *dog* | *M - F* | 4.57 | 0.31 |
| *Layla-Zuri* | *dog* | *F -F* | 6.43 | 0.28 |
